# Effect of Cosolutes on the Sorption of Phenanthrene onto Mineral Surface of River Sediments and Kaolinite

**DOI:** 10.1155/2014/812531

**Published:** 2014-07-22

**Authors:** Yinghong Wu, Fang Liu, Wen Zhang, Lei Wang

**Affiliations:** ^1^Tianjin Centers for Disease Control and Prevention, Tianjin 300171, China; ^2^Ministry of Education Key Laboratory of Pollution Processes and Environmental Criteria/Tianjin Key Laboratory of Environmental Remediation and Pollution Control, Nankai University, Tianjin 300071, China; ^3^Key Laboratory for Applied Microbiology of Shandong Province, Biology Institute of Shandong Academy of Sciences, Jinan 250014, China

## Abstract

Sorption of phenanthrene onto the natural sediment with low organic carbon content (OC%), organic-free sediment, and kaolinite was investigated through isotherm experiments. Effects of cosolutes (pyrene, 4-n-nonyphenol (NP), and humic acid (HA)) on phenanthrene sorption were also studied by comparing apparent solid-water distribution coefficients (*K*_*d*_^app^) of phenanthrene. Two addition sequences, including “cosolute added prior to phenanthrene” and “cosolute and phenanthrene added simultaneously,” were adopted. The Freundlich model fits phenanthrene sorption on all 3 sorbents well. The sorption coefficients on these sorbents were similar, suggesting that mineral surface plays an important role in the sorption of hydrophobic organic contaminants on low OC% sediments. Cosolutes could affect phenanthrene sorption on the sorbents, which depended on their properties, concentrations, and addition sequences. Pyrene inhibited phenanthrene sorption. Sorbed NP inhibited phenanthrene sorption at low levels and promoted sorption at high levels. Similar to NP, effect of HA on phenanthrene sorption onto the natural sediment depended on its concentrations, whereas, for the organic-free sediment and kaolinite, preloading of HA at high levels led to an enhancement in phenanthrene *K*_*d*_^app^ while no obvious effect was observed at low HA levels; dissolved HA could inhibit phenanthrene sorption on the two sorbents.

## 1. Introduction

Sorption of polycyclic aromatic hydrocarbons (PAHs) to suspended and settled sediments has a major influence on their transport, bioavailability, and fate in natural aquatic environment. Soil/sediment organic matter (SOM) is usually believed to be the principle sorbing component for PAHs and other hydrophobic organic chemicals (HOCs) [1–3]. However, contribution of mineral surface to the sorption of HOCs cannot be ignored, especially for the sorbents with low content of SOM [4–7].

Organic contaminants commonly coexist in natural aquatic environment, and cosolutes were found to affect the sorption process of the target pollutant [8, 9]. In addition, these cosolutes do not always enter the natural aquatic environment simultaneously with the target pollutant and their contacting sequence with the sorbents is also a considerable factor for the sorption of HOCs in coexisting systems [10]. For example, appreciable sorption completion from naphthalene was observed for phenanthrene presorbed on soil, while the competition was little when naphthalene and phenanthrene contacted soil simultaneously [11].

In this study, phenanthrene, a widespread three-ringed PAH, was selected as a representative HOC, and its sorption on the natural sediment with low organic carbon content (OC%), organic-free sediment, and kaolinite was investigated. Pyrene, 4-n-nonylphenol (NP), and humic acid (HA) were chosen as representative coexisting PAH, polar organic pollutant, and DOM, respectively, to study their effects on phenanthrene sorption onto the three sorbents. Pyrene is a typical four-ringed PAH, which is also widely observed in the environments. NP was selected because of its widespread occurring in the world as an intermediate from degradation of nonylphenol ethoxylates (NPEOs), a widely used nonionic surfactant [12]. Two addition sequences, including “cosolute added prior to phenanthrene” and “cosolute and phenanthrene added simultaneously,” were adopted. The objectives of this study were (i) to understand the role of the mineral surface of the sorbents in phenanthrene sorption and (ii) to examine the effects of 3 kinds of cosolutes on phenanthrene sorption.

## 2. Materials and Methods

### 2.1. Sorbents and Sorbates

The natural sediment was collected from the top 0–20 cm of the sediment in Lanzhou Reach of the Yellow River. It was washed using double distilled water, air-dried, and sieved to obtain the size fraction of 38–75 *μ*m. H_2_O_2_ treatment was performed to obtain the organic-free sediment according to Kunze and Dixon [13], followed by the steps of washing, drying, and sieving. The same size fraction as the natural sediment was used for the experiment. Kaolinite (a product of inorganic clay) was purchased from Fuchen Chemical Reagent Co., Inc. (Tianjin, China) and was used without further treatment.

Phenanthrene and pyrene were purchased from Acros Organics (New Jersey, USA). NP was purchased from Tokyo Chemical Synthesis Ind. Co. Ltd (Tokyo, Japan). They were dissolved in methanol to make their respective stock solution. Humic acid (HA) was purchased from Tianjin Guangfu Fine Chemical Research Institute in China, which was dissolved in 0.1 mM NaOH solution and filtrated through a 0.45 *μ*m membrane to obtain a stock solution (0.89 g/L).

### 2.2. Sorption Experiments

Sorption isotherms of phenanthrene on the three sorbents (natural sediment, organic-free sediment, and kaolinite) were determined using a batch equilibration method. Fifty milligrams of the sorbent was weighed into 10 mL glass centrifuge tubes. Background solution (9 mL) was added into the tubes, which contained 1 mM CaCl_2_ *·*2H_2_O, 0.1 mM MgCl_2_, 0.5 mM Na_2_B_4_O_9_ *·*10H_2_O, and 200 mg/L NaN_3_ as bacteria inhibitor [14]. Proper amount of phenanthrene stock solution was spiked to make sure that the initial phenanthrene concentration was in the range of 0.1–2 mg/L and the volume percentage of methanol in each vial was kept below 0.2% (v/v). The tubes were sealed with grounding glass caps and Teflon liners. All tubes were hand-shaken for a few minutes to make them uniformly mixed and then horizontally placed in a 25°C air bath shaker oscillated at 150 rpm for 24 h, during which our preliminary test showed that apparent sorption equilibrium occurred (see Figure S1 in Supplementary Material available online at http://dx.doi.org/10.1155/2014/812531). The vials were centrifuged at 4800 rpm for 20 min after the sorption experiment. The supernatants were analyzed using high-performance liquid chromatography (HPLC). Loss of phenanthrene due to photochemical decomposition, evaporation, and sorption to vials was less than 2%. Sorbed phenanthrene concentrations on the sorbents were calculated by mass balance.

To study the effect of the cosolutes (pyrene, NP, and HA), experiments were divided into two groups according to different adding sequence of cosolutes.* Group 1*: the cosolute was added into the above solid-water system at different concentrations, shaken for 24 h at 150 rpm, and then centrifuged (4800 rpm, 20 min). The supernatant was replaced by 8 mL of background solution containing 1.0 mg/L of phenanthrene and the vials were then shaken for 24 h.* Group 2*: the cosolute was added immediately after phenanthrene was added to the above solid-water system. The coexisting systems were shaken for 48 h. The vials were centrifuged and the supernatants were analyzed. All experiments were conducted in triplicate.

### 2.3. Chemical Analysis

HPLC (Waters 1525, USA) equipped with a fluorescence detector (Waters 2475, USA) and a reverse-phase column (C_18_ column, *μ*Bondapak 3.9 mm i.d. × 300 mm × 10 *μ*m, Waters, USA) was utilized for analysis of phenanthrene, pyrene, and NP. The mobile phase was acetonitrile: water (80 : 20, v/v) and the flow rate was 1.0 mL/min. Excitation/emission wavelengths for fluorescence detector were 280/355 nm for phenanthrene, 333/390 nm for pyrene, and 233/302 nm for NP. All measurements were conducted in duplicate with the uncertainty less than 5%. The detection limits were 0.50 *μ*g/L for phenanthrene, 0.30 *μ*g/L for pyrene, and 0.05 mg/L for NP. HA concentration in water solution was determined using a UV-Visible spectrophotometer (Varian Cary 50 Conc., USA) at 254 nm according to Lu and Wu [15]. A surface area analyzer (ASAP2010, Micromeritics Instrument Corporation, USA) was used to determine the Brunaer-Emmett-Teller (BET) specific surface area and porosity of the three sorbents by application of the multipoint adsorption isotherms of N_2_. Organic carbon (OC) content of the three sorbnets was determined using Walkley-Black method [16].

## 3. Results and Discussion

### 3.1. Properties of the Sorbents

**Figure 1:**
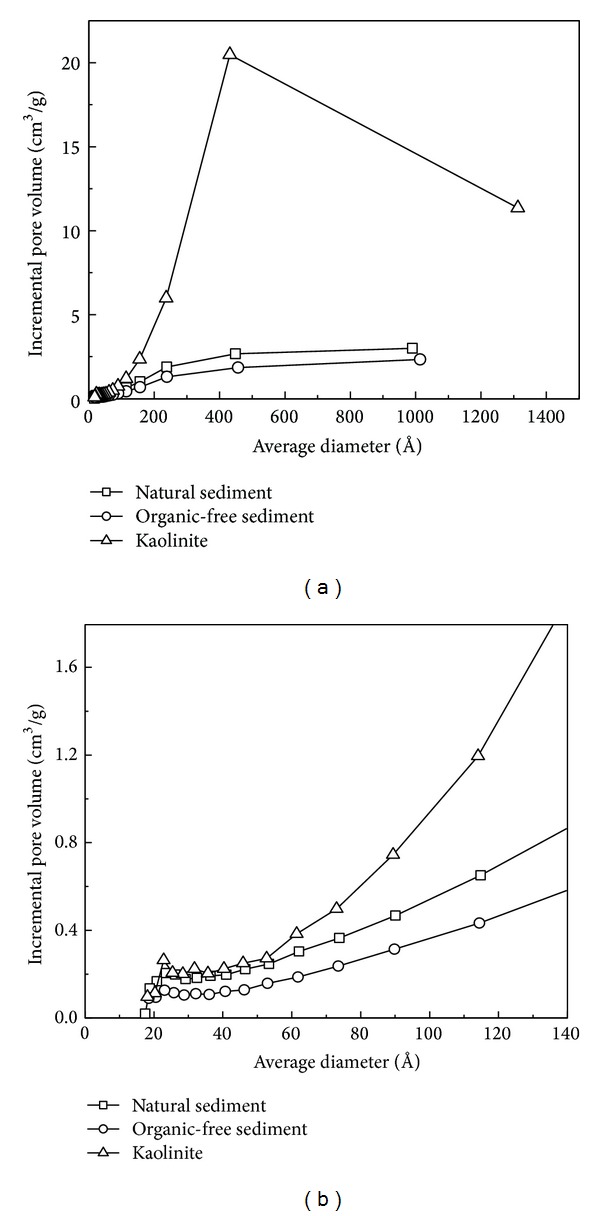
Pore volume distribution of three sorbents: (a) with average diameter ranging in 0–1400 Å; (b) with average diameter ranging in 0–140 Å.

Selected properties of the sorbents, including OC content, specific surface area, and porosity are shown in Table 1. The OC content of the natural sediment used in this study was 0.27%, which is much lower than those of the sediments in other areas (1.2%–11%) [17, 18]. The natural sediment possessed intermediate BET surface area (4.68 m^2^/g) and pore volume (1.26 × 10^−2^ cm^3^/g) among the three sorbents. H_2_O_2_ treatment decreased its OC content as expected, with 0.06% of OC in the treated sediment (organic-free sediment). Its BET surface area and pore volume were also decreased (BET surface area: 2.97 m^2^/g; pore volume: 0.85 × 10^−2^ cm^3^/g). It suggests that NOM of the natural sediment contributed much to the BET surface area and pore volume. Kaolinite BET surface area and pore volume were much larger than those of the other two sorbents, which could be ascribed to the large inner surface of its lamellar structure. The pore volume distributions of the three sorbents are presented in Figure 1. The pores larger than 40 nm in width were predominant for all the three sorbents, especially for the kaolinite.

### 3.2. Phenanthrene Sorption on the Sorbents

**Figure 2:**
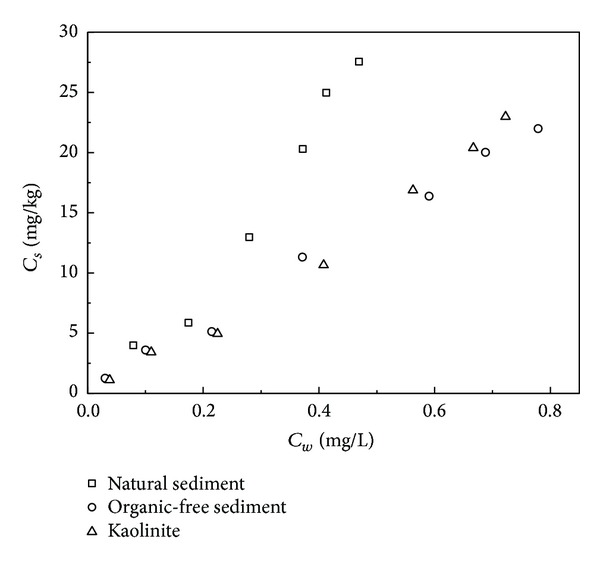
Sorption isotherms of phenanthrene on 3 sorbents.

The results of the sorption isotherms show that there was no significant difference in phenanthrene sorption on the 3 sorbents (Table 2, Figure 2). Freundlich and linear equations were adopted to describe the sorption of phenanthrene and Freundlich model fits the isotherm data better (Table 2). The log⁡ *K*_*F*_ of phenanthrene for the natural sediment and kaolinite is similar, with the value of 1.50. For the organic-free sediment, a little lower log⁡*K*_*F*_ (1.45) was gained. The slight difference in log⁡*K*_*F*_ between the natural sediment and the organic-free sediment might be attributed to the difference in their *f*_oc_, which is important for HOCs sorption. Sun and Yan studied the roles of different parts of NOM in HOCs sorption [19] and found humic acid and humin controlled sorption and humin was the key factor. Oxidation could not affect the content of humin, which could explain the similar *K*_*F*_ of natural sediment and its organic-free sorbent. Furthermore, the difference in log⁡ *K*_*F*_ was not great, which means that NOM plays limited role in the sorption of phenanthrene on these low OC% sediments and mineral surface contributes much to the sorption process. The sorption capability of kaolinite was similar to that of the natural sediment, even if it possesses larger specific surface area and pore volume. Pore volume was found important to the sorption of PAHs on solids [20]. However, kaolinite would form surface hydration on its hydrophilic mineral surface, making sorption sites less accessible to phenanthrene molecules [7].

### 3.3. Effect of Cosolutes on Phenanthrene Sorption

#### 3.3.1. Effect of Pyrene

**Figure 3:**
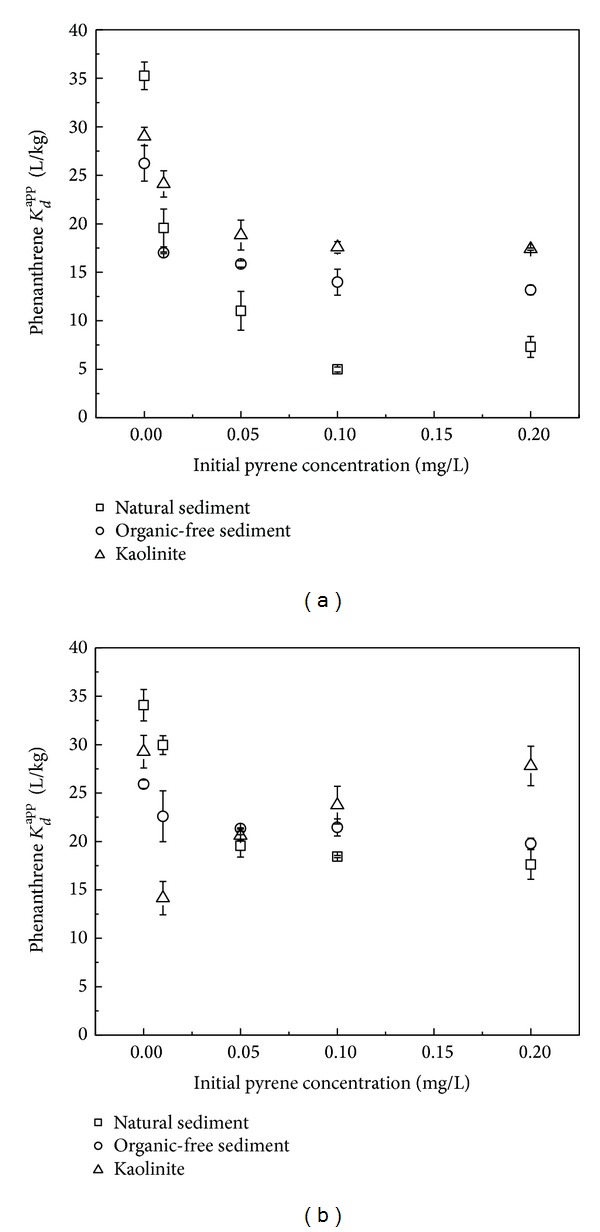
Effect of pyrene on *K*_*d*_^app^ of phenanthrene. (a) Group 1; (b) Group 2.

Effect of pyrene on *K*_*d*_^app^ of phenanthrene is shown in Figures 3(a) and 3(b). In Group 1 (Figure 3(a)), pyrene was added into the solid-water system and reached equilibration; its aqueous solution was partially replaced by phenanthrene solution. The *K*_*d*_^app^ of phenanthrene for the three sorbents decreased markedly with the increased addition of pyrene. For the natural sediment, the addition of 0.20 mg/L pyrene decreased the *K*_*d*_^app^ of phenanthrene from 35.2 L/kg to less than 10.0 L/kg, while for organic-free sediment and kaolinite, the corresponding *K*_*d*_^app^ decreased from 26.2 and 29.0 L/kg to 13.1 and 17.3 L/kg, respectively. Pyrene attenuated phenanthrene sorption because it was expected to occupy the sorption sites during the first 24 h and in the latter 24 h the residual still possibly competed for these sites after phenanthrene was introduced.

In Group 2 (Figure 3(b)), pyrene was added almost simultaneously with phenanthrene. The inhibition effect of pyrene on phenanthrene sorption was fainter than in Group 1. In the presence of 0.20 mg/L pyrene, Δ*K*_*d*_^app^ of phenanthrene for the natural sediment, organic-free sediment, and kaolinite was 16.5, 6.1, and 1.5 L/kg, respectively (Group 1: 28.0, 13.0, and 11.6 L/kg). This difference could be attributed to the accessibility of sorption sites to phenanthrene molecules in the presence of pyrene. The diffusivity of the larger pyrene molecule in water is lower than that of the phenanthrene molecule; thus, the phenanthrene molecule is expected to get into contact with the sorbent earlier.

For Groups 1 and 2, the *K*_*d*_^app^ of phenanthrene decreased quickly at low initial pyrene concentrations; with the initial pyrene concentration increasing, the dropping trend of phenanthrene *K*_*d*_^app^ became gentle, and for some sorbent it even reversed. It is possibly because pyrene in solution decreases the solubility of phenanthrene due to the similar molecular structure, which is in favor of phenanthrene sorption and against the decrease in phenanthrene *K*_*d*_^app^. Meanwhile, codissolved pyrene still competed for sorption sites, which accounted for the observed decrease in *K*_*d*_^app^ values. The cumulative effect was the balance of the two aspects. Sorption on kaolinite in Group 2 was interesting. The *K*_*d*_^app^ of phenanthrene first decreased to a minimum (29.3 to 14.1 L/kg) at 0.01 mg/L of initial pyrene concentration and it began to increase (≥0.05 mg/L) and finally reached 27.8 L/kg at 0.20 mg/L. However, such increase was not observed for kaolinite in Group 1, which was supposed to have greater increase in phenanthrene *K*_*d*_^app^ since there was enough time for pyrene to occupy sites preferentially. It will require further research to identify the mechanisms involved.

#### 3.3.2. Effect of NP

**Figure 4:**
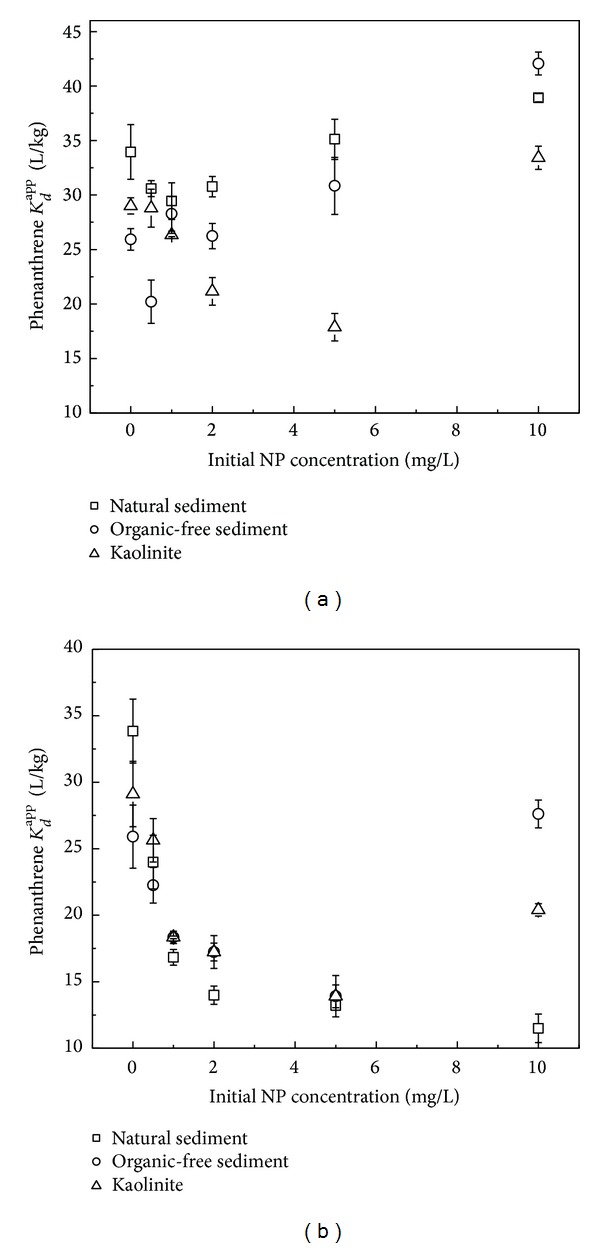
Effect of NP on phenanthrene *K*_*d*_^app^ (a) Group 1; (b) Group 2.

Effect of NP on *K*_*d*_^app^ of phenanthrene is shown in Figures 4(a) and 4(b). In Group 1, with NP initial concentration increasing, the *K*_*d*_^app^ of phenanthrene decreased at low NP levels and changed to increase at high levels, which is similar to the effect of pyrene on phenanthrene *K*_*d*_^app^. When initial NP concentration reached a maximum (10 mg/L) in this study, phenanthrene *K*_*d*_^app^ on the natural sediment, organic-free sediment, and kaolinite was 38.9, 42.1, and 33.4 L/kg, respectively. These values surpassed phenanthrene *K*_*d*_^app^ in the system without NP (34.0, 25.9, and 29.0 L/kg, resp.), which is different from the effect of pyrene.

Inhibition on HOC sorption by low concentrations of surfactant has been observed in a few studies, which was considered to result from the competition for hydrophobic sorption sites between the surfactant and phenanthrene molecules [8, 21]. However, the hydrophobic sites of the sorbents used in this study were believed to be deficient, especially for the organic-free sediment and kaolinite. Space hindrance is thus a possible cause of the inhibition of phenanthrene sorption. Figure S2 in Supplementary Material shows a scheme of successive adsorption stages of a surfactant on the hydrophilic surface [22]. At low surfactant levels, the surfactant on the sorbent surface exists as stages I and II, so that space hindrance plays an important role in reducing the accessibility of phenanthrene to active adsorption sites on the sorbent surface. With more surfactants onto the surface (stage III), the adsorbed surfactant appears to form a thin organic film (stages IV and V), which can effectively absorb phenanthrene and result in high *K*_*d*_^app^. Enhancement in HOC sorption by surfactants has been reported before. Edwards proposed that the sorbed surfactant enhanced HOC sorption not only through increasing OC content of the sorbent, but also acting as an effective sorbent for HOCs [23]. With more surfactant molecules sorbing onto the solids, the hydrophobic tails of the sorbed surfactants are displaced from the surface by hydrophobic groups of the adjacent molecules. Hence, the hydrophobic tails of the sorbed surfactants get more chances to interact with the HOC solutes, which could enhance HOC sorption through bridging action [22]. Accordant result has been observed by Zhu et al. [24]. In addition, when NP concentration increased to some extent, micelle or semimicelle might form on the mineral surface, even if the solution concentration of NP was significantly lower than its critical micelle concentration (CMC, about 6 × 10^−5^ mol/L[25]). Therefore, partitioning of HOC molecule to surfactant phase on the surface might play a predominant role in their sorption process [8]. 

In Group 2, with the increased addition of NP, the *K*_*d*_^app^ of phenanthrene decreased; at the highest initial NP concentration (10 mg/L), the trend of phenanthrene *K*_*d*_^app^ on the organic-free sediment and kaolinite was reversed. In the solution containing 10 mg/L of NP, the *K*_*d*_^app^ of phenanthrene on the natural sediment, organic-free sediment, and kaolinite was 11.5, 27.6, and 20.4 L/kg, respectively, which were much lower than those in Group 1. It is probably because large amount of NP (5-6 mg/L) still existed in water at the end of the experiment in Group 2, which enhanced phenanthrene water solubility and decreased its *K*_*d*_^app^, but most of these dissolved NP has been adsorbed onto the sorbents and removed from water when NP was added previously in Group 1.

#### 3.3.3. Effect of HA

**Figure 5:**
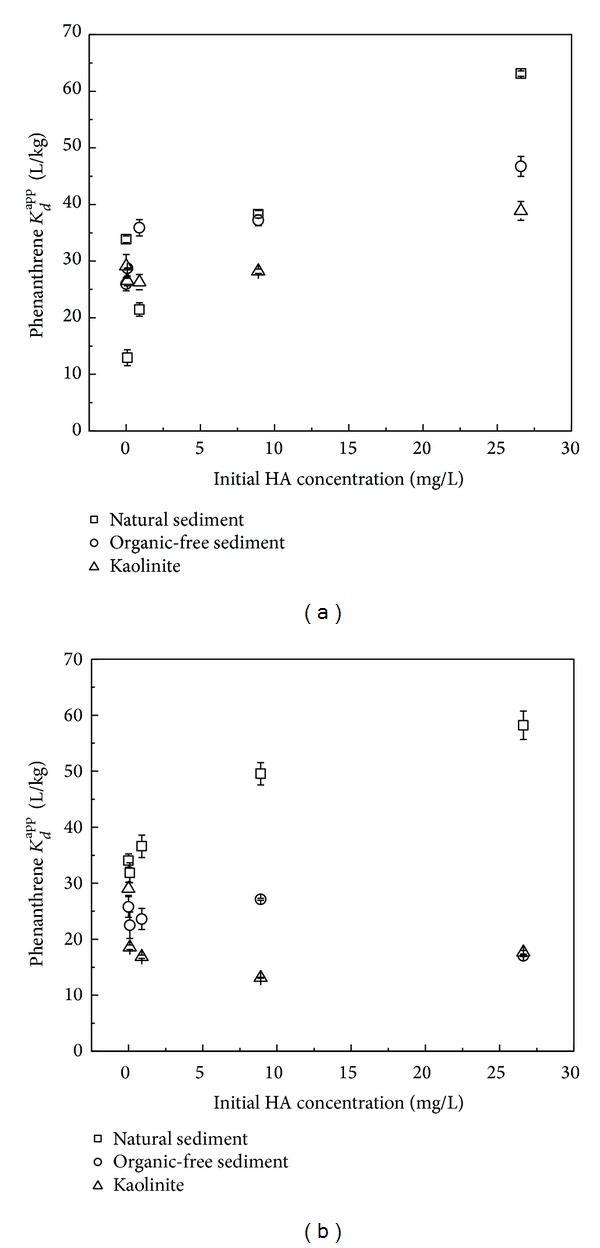
Effect of HA on phenanthrene *K*_*d*_^app^ (a) Group 1; (b) Group 2.

Figures 5(a) and 5(b) show the effect of dissolved HA on the sorption of phenanthrene on the three sorbents. In Group 1, for the natural sediment, the effect of HA depended on its addition amount, just like the effect of NP. Inhibition of phenanthrene sorption on the natural sediment was observed at relatively low HA concentrations, which could be attributed to the competition between phenanthrene and HA for the adsorption sites. By competing with the active sites on the mineral surface, sorbed HA might decrease the sorption of phenanthrene on the sorbents, especially for the irreversible sorption domain. Great enhancement for phenanthrene sorption was observed at high HA concentrations, with phenanthrene *K*_*d*_^app^ increasing from 33.8 L/kg (without HA) to 63.0 L/kg (HA: 26.6 mg/L). This enhancement should be explained by the increase of particle OC content or hydrophobic sorption sites due to sorption of HA on the surface of the natural sediment, which should mainly belong to reversible sorption domain. Previous sorption of HA at high levels also led to an enhancement in phenanthrene sorption onto organic-free sediment and kaolinite, with Δ*K*_*d*_^app^ of 20.8 and 9.8 L/kg, respectively, while no obvious effect was observed at low HA levels. The difference in the effect of HA on phenanthrene *K*_*d*_^app^ among the three sorbents is believed to result from their different physical and chemical properties, which could control HA sorption and could also be modified by HA in turn, affecting phenanthrene sorption.

In Group 2, for the natural sediment, HA addition led to a continuous increasing *K*_*d*_^app^ of phenanthrene, which reached 58.2 L/kg at a maximum level of HA in this study (26.6 mg/L). However, HA inhibited the sorption of phenanthrene on the organic-free sediment and kaolinite, and the *K*_*d*_^app^ decreased to less than 20.0 L/kg. It is probably because for the two sorbents lacking of OC, HA was difficult to be adsorbed and dissolved HA in solution seems to predominate in phenanthrene sorption. The partition coefficients of phenanthrene between HA and water on an organic carbon basis (log⁡*K*_DOC_) were usually between 3.6 and 3.9 [26]; thus, DOM-bound HOC could be the dominant speciation of HOCs in DOM solution. DOM residue in solution could thus enhance the apparent solubility of HOCs [27, 28], which affects their distribution.

## 4. Conclusions

Phenanthrene sorption on the natural sediment with low OC%, organic-free sediment, and kaolinite was similar, suggesting that mineral surface can play an important role in the sorption of hydrophobic organic contaminants on low OC% sediments. Cosolutes affected phenanthrene sorption on these sorbents, whose properties, concentrations, and addition sequences are key factors. Pyrene inhibited phenanthrene sorption. The *K*_*d*_^app^ of phenanthrene decreased quickly at low initial pyrene concentrations and gently increased at high concentrations. Sorbed NP inhibited phenanthrene sorption at low levels and promoted sorption at high levels; in addition, high levels of dissolved NP could decrease phenanthrene *K*_*d*_^app^ by enhancing phenanthrene water solubility. Effect of HA on phenanthrene sorption onto the natural sediment depended on its concentrations, just like NP, whereas, for the organic-free sediment and kaolinite, preloading of HA at high levels led to an enhancement in phenanthrene *K*_*d*_^app^ while no obvious effect was observed at low HA levels; dissolved HA could inhibit phenanthrene sorption on the two sorbents.

## Supplementary Material

Two figures, i.e. Figures S1 and S2, were included in the Supplementary Material. Figure S1 showed the result of kinetics experiment. Figure S2 cited a graphic to explain the state of sorbed nonylphenol on the mineral surface.

## Figures and Tables

**Table 1:** Properties of the sorbents.

Sorbents	BET area (m^2^/g)	Pore volume (10^−2^ cm^3^/g)	OC (%)
Natural sediment	4.68 ± 0.02	1.26	0.27
Organic-free sediment	2.97 ± 0.03	0.85	0.06
Kaolinite	8.05 ± 0.10	4.50	0.08

**Table 2:** Isotherm parameters of phenanthrene sorption on the sorbents.

Sorbents	Freundlich model			Linear model
	log⁡*K*_*F*_^a^	*n*	*R* ^2^	*R* ^2^
Natural sediment	1.50 ± 0.02^b^	1.250 ± 0.05	0.976	0.930
Organic-free sediment	1.45 ± 0.02	0.883 ± 0.02	0.986	0.994
Kaolinite	1.50 ± 0.08	1.017 ± 0.01	0.986	0.976

^a^Unit: *K*_*F*_ ((mg/kg)/(mg/L)^*n*^); ^b^Standard error.
