# Supplementary material for: Effect of Cosolutes on the Sorption of Phenanthrene onto Mineral Surface of River Sediments and Kaolinite
Source: ScientificWorldJournal. 2014 Jul 22;2014:812531. doi: 10.1155/2014/812531 (PMC4132337; doi:10.1155/2014/812531)
Supplement: Supplementary file 1 — Two figures, i.e. Figures S1 and S2, were included in the Supplementary Material. Figure S1 showed the result of kinetics experiment. Figure S2 cited a graphic to explain the state of sorbed nonylphenol on the mineral surface. [file 812531.f1.pdf]

Supporting information for:

Effect of Cosolutes on the Sorption of Phenanthrene onto Mineral Surface of River Sediments and Kaolinite

Yinghong Wu[1,*], Fang Liu[2], Wen Zhang[3], Lei Wang[2]

[1] Tianjin Centers for Disease Control and Prevention, Tianjin 300171, China

[2] Ministry of Education Key Laboratory of Pollution Processes and Environmental Criteria/Tianjin Key Laboratory of Environmental Remediation and Pollution Control, Nankai University, Tianjin 300071, China; E-Mail: liufang900520@163.com (F.L.); L.wang@live.com (L.W.);

[3] Key Laboratory for Applied Microbiology of Shandong Province, Biology Institute of Shandong Academy of Sciences, Jinan 250014, China; E-Mail: zw-sunshine@163.com (W.Z.)

* Author to whom correspondence should be addressed; E-mail: wuyinghongnk@126.com;

Tel: +86-22-23504362; Fax: +86-22-23504362.

For submission to: The Scientific World Journal

Figures:2
Pages: 3

Fig. S1

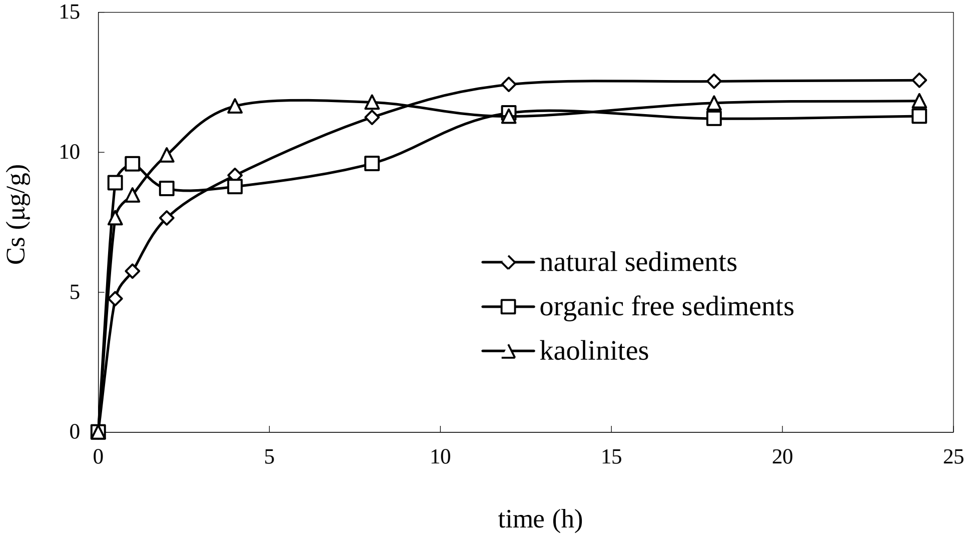

Figure S1. Sorption kinetic of phenanthrene on 3 sorbents

Fig. S2

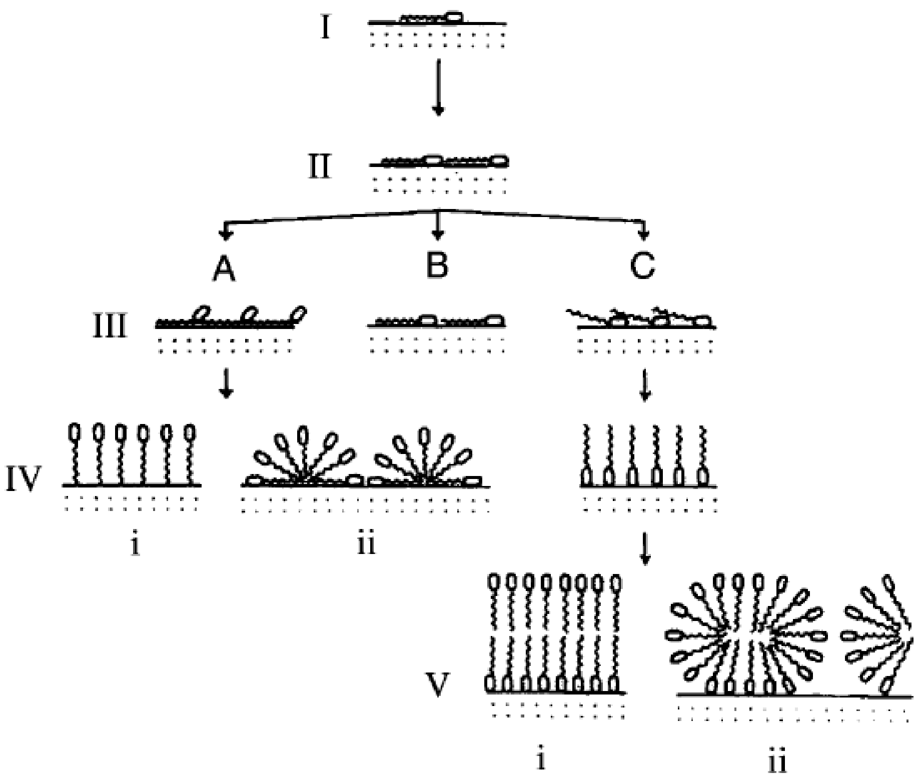

Figure S2. Successive stages of adsorption of non-ionic surfactant. Reproduced from the following reference.

Ref: PARIA S., KHILAR K. C. A review on experimental studies of surfactant adsorption at the hydrophilic solid-water interface. Adv. Colloid Interfac. 2004, 110, 75-95.
